# AlphaFill: enriching AlphaFold models with ligands and cofactors

**DOI:** 10.1038/s41592-022-01685-y

**Published:** 2022-11-24

**Authors:** Maarten L. Hekkelman, Ida de Vries, Robbie P. Joosten, Anastassis Perrakis

**Affiliations:** Oncode Institute and Department of Biochemistry, The Netherlands Cancer Institute, Amsterdam, the Netherlands

**Keywords:** Protein databases, Protein structure predictions

## Abstract

Artificial intelligence-based protein structure prediction approaches have had a transformative effect on biomolecular sciences. The predicted protein models in the AlphaFold protein structure database, however, all lack coordinates for small molecules, essential for molecular structure or function: hemoglobin lacks bound heme; zinc-finger motifs lack zinc ions essential for structural integrity and metalloproteases lack metal ions needed for catalysis. Ligands important for biological function are absent too; no ADP or ATP is bound to any of the ATPases or kinases. Here we present AlphaFill, an algorithm that uses sequence and structure similarity to ‘transplant’ such ‘missing’ small molecules and ions from experimentally determined structures to predicted protein models. The algorithm was successfully validated against experimental structures. A total of 12,029,789 transplants were performed on 995,411 AlphaFold models and are available together with associated validation metrics in the alphafill.eu databank, a resource to help scientists make new hypotheses and design targeted experiments.

## Abstract (web summary)

The AlphaFill algorithm transplants missing small molecules and ions from experimentally determined structures to predicted protein models in the AlphaFold protein structure database. All AlphaFill entries are available for visual inspection and download through the AlphaFill website.

## Main

Predicting the three-dimensional (3D) structure of a protein based on its amino-acid sequence alone has been a major scientific challenge for decades. Recently, artificial intelligence approaches, as implemented in the AlphaFold^1^ and the RoseTTAfold^2^ methods, have made protein structure prediction unprecedently reliable. Both approaches predict domain structures with impressive accuracy, but flexible parts of the protein (such as loops or intrinsically disordered regions) are understandably predicted with lower accuracy and confidence. Predictions for the proteomes of 48 different organisms, as well as all SWISS-PROT^3^ entries, have been publicly available in the AlphaFold protein structure database^4^—about a million predicted protein structures—at the time of this study, and more than 200 million followed in July 2022. These predicted models are already providing invaluable new biological insights regarding protein function.

The artificial intelligence prediction algorithms have not been trained to solve the protein folding problem from first principles. They have merely, yet impressively, learned the inherent rules of protein folding based on extensive training on experimentally resolved structures. However, many proteins do not occur in nature without their cofactor: myoglobin or hemoglobin need a heme to fold; zinc-finger domains are not stable without a zinc ion and many proteins can only exist as homo- or hetero-multimers^5^. The multimer issue was addressed by the development of AlphaFoldMultimer^6^ and RoseTTAFold^7^, that can predict complex protein assemblies. However, predicted structural models exclusively account for the 20 canonical amino-acid residues, and do not predict the coordinates for small molecules, ligands and cofactors typically associated with a protein.

Here, we enrich the models in the AlphaFold database by ‘transplanting’ small molecules and ions that have been experimentally observed in homologous protein structures. The AlphaFill procedure we present has been validated against experimental structures and applied to all AlphaFold models to create a new resource, the AlphaFill databank, which is designed to help life scientist to easily generate new hypotheses for protein function and formulate relevant research questions.

## Results

### Transplanting compounds to AlphaFold models

First, we search for sequence homologs for each structure in the AlphaFold database in the PDB-REDO databank^8^. We consider structures with identity higher than 25% over an aligned sequence of at least 85 residues as hits. The most common ligands in the PDB, as well as cofactors and their analogs from the CoFactor database^9^ are kept as candidates for the ‘transplants’. Currently, we are transplanting 2,694 different compounds that represent over 95% of all ligand occurrences in the Protein Data Bank (PDB)^10^.

Next, the selection of structures with compounds of interest are structurally aligned^11^ on the Cα-atoms of the AlphaFold model, and the root-mean-square deviation (r.m.s.d.) is calculated (global r.m.s.d.). Starting from the closest homolog, all backbone atoms within 6 Å from the atoms of each compound that will be considered for ‘transplantation’ are selected and used for a local structural alignment to the AlphaFold model; the r.m.s.d. from this alignment is also calculated (local r.m.s.d.). Compounds are then transplanted into the AlphaFold model to make the AlphaFill model, unless the same compound has already been placed within 3.5 Å of the centroid of the compound to be fitted (originating from a previously considered homolog). All AlphaFill models and metadata are stored in the AlphaFill databank.

Further details on the procedure are available in the Methods.

### The AlphaFill databank

Applying the AlphaFill approach to the AlphaFold database available in February 2022 (995,411 models) resulted in 586,137 models that had at least one transplanted compound. A total of 12,029,789 compounds were transplanted into these models. A selection of frequently transplanted compounds is listed in Table 1, including their ‘transplantation’ frequency at four levels of sequence identity (25, 30, 50 and 70%), which we chose empirically. The numbers for all transplanted compounds at 25, 30, 40, 50, 60 and 70% are available from the AlphaFill website.

**Table 1:** Examples of frequently transplanted compounds in the AlphaFill databank for indicative levels of sequence identity: trans., transplants

Sequence identity		25%		30%		50%		70%	
Compound code and name		No. of entries	No. of transplants	No. of entries	No. of transplants	No. of entries	No. of transplants	No. of entries	No. of transplants
**Nucleotides**									
ADP	Adenosine diphosphate	100,258	242,131	77,591	166,420	26,804	42,189	10,076	13,975
AMP	Adenosine monophosphate	59,639	102,608	44,548	68,972	12,811	18,334	3,951	4,881
ATP	Adenosine triphosphate	67,807	119,001	51,155	83,267	14,729	22,765	5,226	7,223
GDP	Guanosine diphosphate	30,839	77,253	23,810	51,240	10,986	16,702	4,831	6,353
GTP	Guanosine triphosphate	18,274	30,586	14,443	23,841	5,139	7,974	2,054	2,896
UDP	Uridine diphosphate	17,717	25,197	11,119	14,091	2,787	3,184	858	1,040
**Cofactors**									
COA	Coenzyme A	19,037	61,080	12,344	40,880	3,162	11,751	1,369	3,109
FAD	Flavin adenine dinucleotide	18,406	50,295	10,851	23,667	3,111	4,564	1,470	1,958
FMN	Flavin mononucleotide	11,892	26,072	7,732	15,929	2,611	4,054	1,225	1,719
GSH	Glutathione	9,764	20,884	7,535	14,186	2,113	3,021	851	1,122
HEM	Heme	18,675	45,968	11,586	28,849	6,000	13,737	4,242	7,850
NAD	Nicotinamide adenine dinucleotide	35,016	82,533	24,284	50,799	8,542	14,186	3,087	4,898
NAI	NADH	17,223	24,848	11,370	15,858	2,881	3,858	796	1,125
NAP	NAD phosphate/NADP	26,467	67,179	18,142	38,576	4,355	8,286	1,577	2,311
NDP	NADPH	21,598	42,241	14,291	26,603	3,660	7,383	1,535	2,937
PLP	Pyridoxal phosphate	13,462	158,684	10,119	94,516	5,016	12,904	2,131	4,978
SAH	*S*-Adenosyl-l-homocysteine	21,121	30,189	15,692	19,778	4,184	5,079	1,399	1,629
SAM	*S*-adenosylmethionine	21,072	32,467	16,239	23,465	4,449	7,361	1,890	2,948
**Miscellaneous**									
CLA	Chlorophyll A	3,505	443,127	3,217	425,290	1,502	171,022	1,375	157,851
CLR	Cholesterol	8,533	53,500	4,310	18,184	532	1,654	339	866
**Metal ions**									
CA	Calcium(^2+^) ion	202,360	759,181	145,813	473,734	40,010	117,321	15,910	47,819
K	Potassium(^1+^) ion	117,813	270,758	86,633	189,961	23,999	51,361	7,239	13,707
MG	Magnesium(^2+^) ion	328,108	1,981,187	264,320	1,576,629	95,618	514,634	33,595	91,445
NA	Sodium(^1+^) ion	272,353	1,067,005	204,482	734,824	57,076	176,645	19,793	53,329
ZN	Zinc(^2+^) ion	186,268	639,282	135,426	417,736	41,808	99,486	16,675	36,315

All AlphaFill models are available from https://alphafill.eu through a web-based user interface. To enable integration of AlphaFill data in other websites, a 3D-Beacons API (https://github.com/3D-Beacons) is implemented, which is already in use to show AlphaFill entries in the PDBe-Knowledge Base^12^. In addition, the whole databank, including all relevant metadata (that is, the JSON format description of all transplants for each AlphaFill model, a JSON schema with a complete description of these files and the current CIF file that describes the compounds that are considered for transfer) can be downloaded through rsync.alphafill.eu.

### Validation of the AlphaFill algorithm

**Fig. 1:**
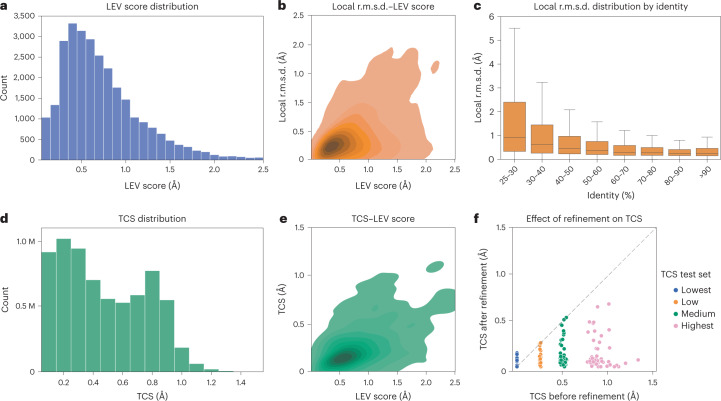
Validation of the AlphaFill algorithm. **a**, Distribution of the LEV score of all transplants obtained with 100% sequence identity (the validation set with *n* = 28,619 independent observations). 408 transplants (1%) with LEV score >2.5 are not shown for clarity. **b**, The local r.m.s.d. correlates with the LEV score in the validation set, Pearson correlation coefficient 0.51 (*n* = 8,039; mono-atomic transplants were not used (main text)). **c**, Distribution of the local r.m.s.d. of all transplants in the AlphaFill models as boxplots in 10% identity ranges. Boxes are based on 3,594,940; 3,866,810; 2,079,705; 1,005,953; 495,357; 369,307; 268,904 and 252,681 transplants, respectively, and extend from first to third quartile with the median as the middle line. Whiskers extend to 1.5 times the interquartile range. For clarity, 332,771; 333,325; 181,126; 79,594; 42,273; 34,634; 29,368 and 24,263 outliers, respectively, are not shown. Maximum values are 107.4, 82.1, 40.6, 37.1, 61.5, 44.4, 35.6 and 35.5 Å. **d**, The distribution of the TCS for all transplants in the AlphaFill models (*n* = 6,859,380). Mono-atomic transplants (5,170,409 compounds) are left out (main text). **e**, The TCS correlates with the LEV score in the validation set (*n* = 8,039; mono-atomic transplants were used (main text)), Pearson correlation coefficient 0.51. **f**, Comparison of the TCS before and after energy minimization for four subsets of the validation set (each with *n* = 50), illustrating that TCS improves for low until highest TCS by refinement.

To validate the AlphaFill algorithm, we compared the transplants created by AlphaFill to experimental structures with 100% sequence identity. We defined the local environment validation (LEV) score as the all-atom r.m.s.d. of any ligand atom and all proteins’ atoms within 6.0 Å from the ligand, between the AlphaFill and experimental complexes. The distribution of the LEV score for all AlphaFill structures within this validation set (28,619 transplants) is presented in Fig. 1a. As the LEV score can be known only when a sequence-identical experimental structure is available, we then compared it to the local r.m.s.d., which we calculate for every transplant as defined above. The LEV score and the local r.m.s.d. correlate well (Fig. 1b). As the local r.m.s.d. can thus be used as a proxy for the quality of each transplant, we analyzed its distribution as a function of sequence identity between the donor and the acceptor model. As expected, local r.m.s.d. goes down with increasing sequence identity (Fig. 1c).

An orthogonal way to validate the quality of a transplant is to evaluate possible clashes between ligand and protein atoms. For this purpose, we defined the transplant clash score (TCS) as a function of the van der Waals overlaps between a transplanted ligand and its binding site (see Methods for details). The distribution of the TCS for all multi-atomic transplants is shown in Fig. 1d. Single atom compounds are overrepresented in the dataset (5,170,409 compounds) and have relatively few clashes, and were thus excluded in evaluating the TCS to avoid biasing the analysis. The TCS correlates well with the LEV score (Fig. 1e). High TCS can suggest an incompatible binding site, suboptimal performance of the AlphaFill algorithm in transplanting the ligand or that the AlphaFold model has local inaccuracies. In the last two cases, clashes could be resolved by local refinement. We thus implemented a procedure using YASARA^13^ to energy minimize a complex. To test this procedure, we chose four sets of 50 complexes each: two sets were defined as the transplants with the lowest and the highest TCS, and two additional categories were chosen around 0.25 and 0.50 Å based on visual inspection of the distribution (Fig. 1d). We then evaluated the TCS before and after energy minimization (Fig. 1f). The TCS slightly increased for some structures in the set with the lowest starting TCS, but is reduced (or unchanged in a few cases) in structures in the other three sets. As the four sets were chosen from the validation set above, we then compared the LEV score before and after energy minimization (Supplementary Fig. 1a). For the lowest and low set, the LEV score is not strongly affected by de-clashing. For medium and highest TCS scores, in many cases the LEV score improves while for others it does not, suggesting that such transplants should be treated with caution.

### Analysis of the quality of AlphaFill databank transplants

The validation was then used to derive quality indicators to annotate the transplants in the AlphaFill databank. As the local r.m.s.d. correlates well with the LEV score (Fig. 1b), we further analyzed its distribution as a function of sequence identity (Fig. 1c) to annotate the transplant. The local r.m.s.d. distribution stays fairly stable for structures with sequence identity of 70% or more (933,117 transplants). We use the values of the local r.m.s.d. exceeding the third quartile plus 1.5 times the interquartile range^14^ for all transplants with sequence identity of 70% or higher (0.92 Å) and for all transplants (3.10 Å) to annotate all AlphaFill transplants as ‘medium confidence’ and ‘low confidence’, respectively (Supplementary Fig. 1b). Using these cutoffs 65.3% of all transplants can be considered high confidence, 24.9% medium confidence and 9.9% low confidence. As the TCS also correlates well with the LEV score (Fig. 1e), we also use it to annotate transplants. Similar to the local r.m.s.d., we used the 1.5 interquartile range cutoff for 70% identity or higher (0.64 Å) and for all transplants (1.27 Å) (Supplementary Fig. 1c), to assign high-confidence (81.3%), medium-confidence (18.6%) and low-confidence (0.05%) transplants based on TCS.

### A web-based user interface for the AlphaFill databank

All AlphaFill entries are available for visual inspection through the AlphaFill website at https://alphafill.eu. On the front page, models can be retrieved using the AlphaFold identifier, which is equivalent to the UniProt primary accession code^15^. Individual entries can also be accessed directly using the same identifier, for example, https://alphafill.eu/?id=P02144 for human myoglobin. The website makes the compound prevalence available (on the Compounds page), as well as numbers of occurrence regarding transplanted compounds for each ‘filled’ AlphaFold model (on the Structures page). The information on the Compounds and Structures pages can be filtered based on sequence identity at cutoffs of 25, 30, 40, 50, 60 and 70%.

**Fig. 2:**
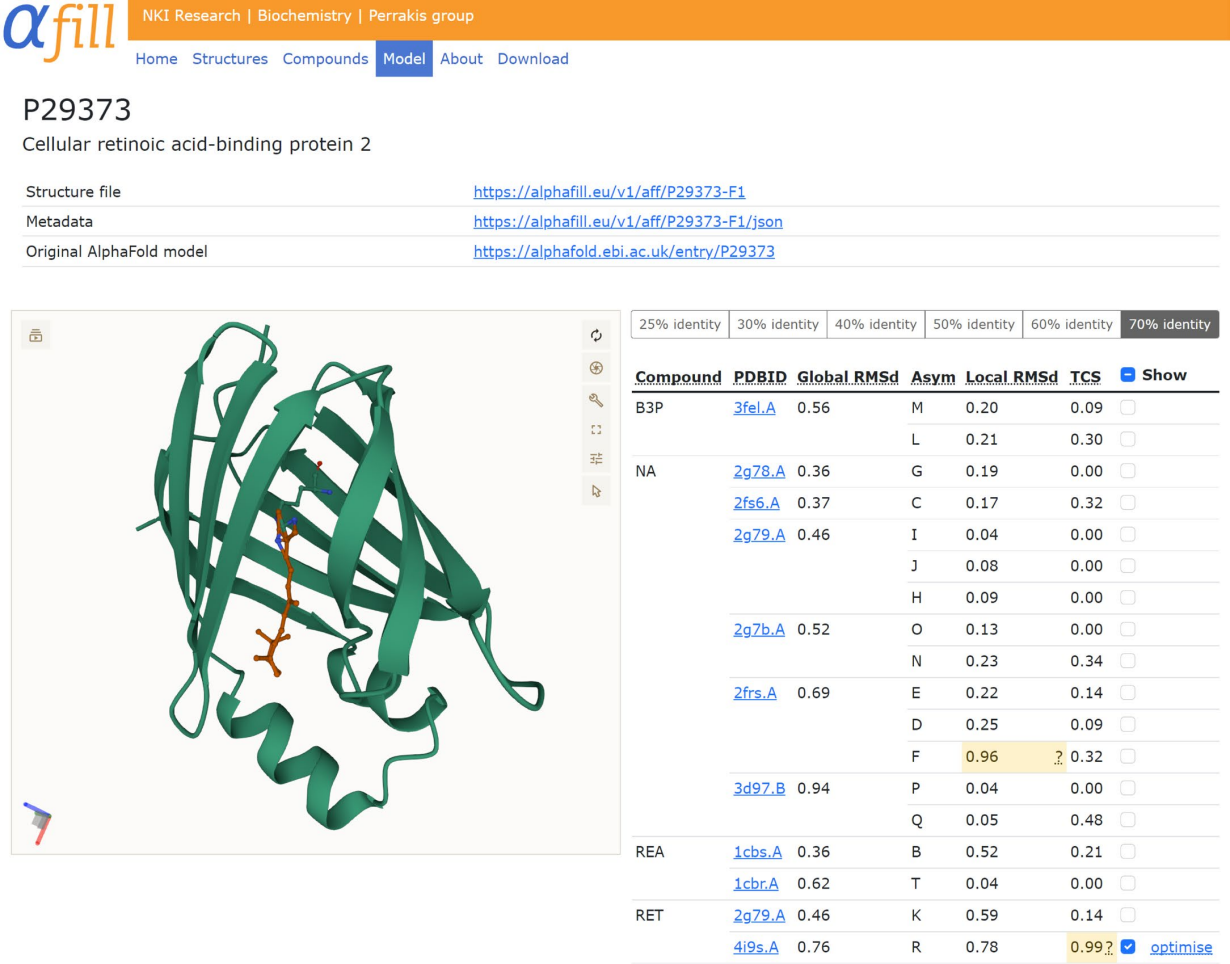
Screenshot of the AlphaFill entry page for cellular retinoic acid-binding protein 2 (AF-P29373). The Mol* viewer on the left can be controlled by the table of transplanted compounds on the right. Clicking a compound in the table brings up a zoom of the binding site. Compounds can be hidden or shown individually using the tick boxes. Transplants at 70% or more sequence identity are displayed. The identity cutoff can be changed using the selector above the table. In this example, retinal (RET) inherited from PDB-REDO entry 4i9s (ref. ^36^) is shown and flagged with a yellow box as medium confidence due to high TCS. All other transplanted compounds are hidden from view, providing the ‘optimize’ option for the selected transplant. After optimization (Supplementary Fig. 2) the is TCS is reduced to 0.29 Å, which is considered high confidence. A sodium from PDB-REDO entry 2frs (ref. ^37^) is flagged for its high local r.m.s.d.

On each entry page (Fig. 2) the selected AlphaFill model is displayed using the visualization software Mol*^16^, allowing users full flexibility for inspection. The ‘transplants’ are listed in a table together with the parent PDB-REDO entry, the global r.m.s.d. between the AlphaFold model and for the hit within the PDB-REDO entry (as a measure of the similarity between the donor and the acceptor structure), the name of the compound (plus the original name if it was mapped), the local r.m.s.d. and the TCS (as quality indicators). Transplants are grouped by compound and sorted by r.m.s.d. (global at the hit level and local at the individual compound level). Clicking a row in the table changes the focus of the viewer to that compound. Compounds can also be toggled on and off to reduce clutter. Transplants are colored in the table by the local r.m.s.d.-based and the TCS-based confidence level (as defined above). Medium-confidence transplants that should be handled with care are marked in yellow; low-confidence transplants requiring caution are marked in red. Using the selector above the table, transplants can be shown at the levels of sequence identity described above. By default, the cutoff is set to the highest identity that displays hits in the table. In practice, this means that if the AlphaFold model can be mapped to an experimental structure with 93% sequence identity, by default only compounds transplanted from structures with more than 70% identity are shown; if only a 28% identical structure exists the default threshold will be set to 25%. When there is no transplant from an experimental structure with greater than 25% identity, the table is blank. A model with all the ligands and the metadata can also be downloaded. If a single transplant is selected in the table, the option to energy minimize (“optimise”) that particular transplant is made available to the user. Following optimization, the TCS score before and after refinement is shown, along with a ligand-focused view (Supplementary Fig. 2), and that particular optimized complex can be downloaded.

## Examples

In the case of models that have identical structures in the PDB, the AlphaFill databank in part reproduces information already in the PDBe-Knowledge Base^12^. However, AlphaFill also transplants compounds from homologous experimental structures that might have been determined in another species, and also to domains for which similar domains are available experimentally. Therefore, the databank offers additional functionality for the annotation of the models that can functionally assist users to make informed decisions about these structures. Here, we will discuss a few examples.

### Myoglobin and heme

**Fig. 3:**
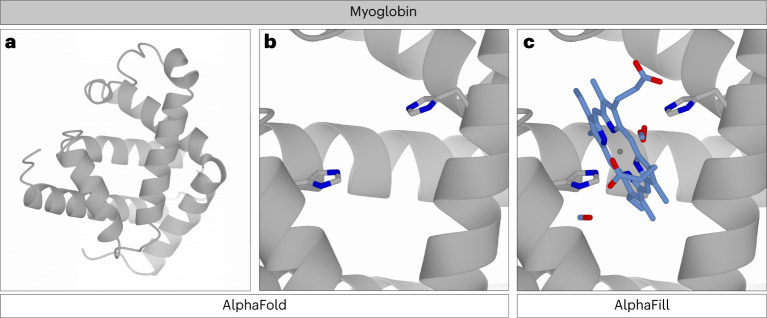
Human myoglobin structures in AlphaFold and AlphaFill. **a**, The ribbon diagram of the AlphaFold model of human myoglobin. **b**, The heme-shaped cavity in the AlphaFold model, wherein the histidine side chains (gray cylinders colored by atom type) are ready to facilitate the heme biding. **c**, The heme-shaped cavity in the AlphaFill model, wherein the binding site is ‘filled’ with the transplanted heme group and the CO and O_2_ ligands; ligands are shown in stick-mode colored by atom type (heme) with the heme iron as a gray sphere.

Human myoglobin is an ɑ-helical protein with heme B as cofactor, binding molecular oxygen and several other small molecules. The AlphaFold model (AF-P02144) is nearly identical to experimentally determined structures, and shows a heme-shaped cavity (Fig. 3). In the AlphaFill databank, many heme analogs (containing metals other than iron) are ‘mapped’ back to heme B (HEM, in PDB nomenclature) based on the data in CoFactor database. The heme analogs 6HE and 7HE that lack a carboxyl tail are not mapped back to heme B, but are instead transferred as is. Additional compounds that are transplanted to the AlphaFold myoglobin model include molecular oxygen and carbon monoxide. The latter is fitted on two locations: one close to the iron atom in heme and the other on the far side of the heme. The second carbon monoxide, located at an unexpected position, is inherited from PDB-REDO entry 1dwt (ref. ^17^), in which it was modeled at 30% occupancy. This occupancy is retained in the AlphaFill model to allow users to take this into account when evaluating the model. The AlphaFill model of myoglobin also contains numerous metal ions. The cobalt and nickel ions should be treated with care as they are inherited from engineered myoglobin dimers (PDB-REDO entries 7dgk and 7dgl, ref. ^18^) that do not have a normal myoglobin fold. This is clearly reflected by the global r.m.s.d. values being above 20 Å.

### Zinc binding sites

The most common transition-metal ion present in macromolecular structures is zinc (Table 1). Typically, it is involved in catalysis or in maintaining structural integrity^19^. The so-called ‘structural zinc ions’ typically involve a tetrahedral binding site containing a combination of four coordinating cysteine and/or histidine residues^20^. As we found before, such tetrahedrals are often distorted in the X-ray models available in the PDB, but the corresponding structures available through PDB-REDO contain improved binding sites^21^ and are better suited for usage in AlphaFill.

**Fig. 4:**
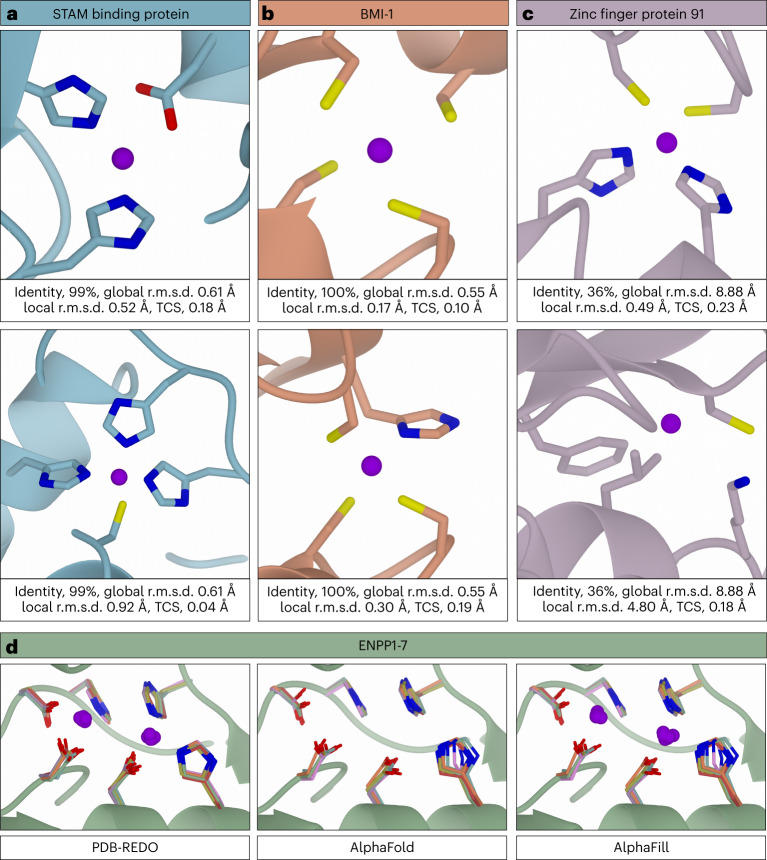
Examples of transplanted zinc ions (purple spheres). All proteins are presented as a ribbon diagram (each protein in a different color, for clarity); side chains coordinating the zinc ions are shown as cylinders colored by atom type for noncarbon atoms. **a**, A catalytic (top) and a structural (bottom) zinc ion in the STAM-binding protein. **b**, Two structural zinc ions in the human BMI-1. **c**, Zinc ion transferred into a structural zinc binding site in the zinc-finger protein 91 (top), wrongly placed zinc ion in the same protein (bottom). **d**, The bimetallic zinc binding site in ENPP1-7 as found in PDB-REDO models (PDB identifiers for ENPP1-7: 6weu, ref. ^38^; 5mhp, ref. ^39^; 6c01, ref. ^40^; 4lqy, ref. ^41^; 5veo, ref. ^42^; 5egh, ref. ^43^ and 5tcd, ref. ^44^, respectively), compared to the same binding site as found in the human ENPP1-7 models from AlphaFold and as available in AlphaFill, containing the two zinc ions. For clarity, only the backbone of ENPP1 is shown as a green ribbon diagram; side chains are colored green, blue, red, pink, orange, purple and gold for ENPP1-7, respectively.

One of the proteins that contains both functional and structural zinc ions is the STAM-binding protein, a zinc metalloprotease that cleaves lysine-63-linked polyubiquitin chains (AF-O95630)^22^. Zinc ions have been transplanted to the AlphaFill model, both at the catalytic site and at the zinc-finger motif (Fig. 4a), originating from the PDB-REDO structure 3rzv (ref. ^22^). The structural zinc ion is coordinated by three histidine residues and one cysteine. Although this tetrahedral zinc binding site looks proper, the atomic distances between the zinc atom and its ligands deviate from previously established target values^21^. This limitation is a consequence of AlphaFold predicting the structure outside the context of key structural elements, in this case the zinc ions. By adding the zinc atom, qualitative information is provided (the zinc atom should be in this binding site), but no quantitative information about the zinc binding site should be extracted from the AlphaFill model. Further refinement of the AlphaFill model with geometric restraints can be applied to make the binding site look more normal.

A similar situation is found for the two ‘transplanted’ zinc ions in the human BMI-1 protein (AF-P35226), which contains two zinc binding sites involved in structural integrity^23^ (Fig. 4b). The binding sites are distorted in terms of coordination geometry with nonoptimal coordination distances and cysteine side chain conformations, but the fact that these are structural zinc binding sites is very clear. The two zinc atoms were transferred by AlphaFill from PDB-REDO entry 3rpg (ref. ^23^), completing the structural overview of BMI-1 with respect to structural integrity.

For ‘zinc-finger protein 91’, an E3 ubiquitin ligase upregulated in prostate cancer, colon cancer and pancreatic cancer^24^, no experimental structures are available, but the human structure is predicted by AlphaFold (AF-Q05481). All transplanted zinc atoms have high global r.m.s.d. values (from 5.71 to 21.87 Å), but many have good local r.m.s.d. and TCS values. One such zinc atom is Zn AB originated in PDB-REDO entry 5wjq (ref. ^25^) (Fig. 4c). The global r.m.s.d. is high (8.88 Å), but the local r.m.s.d. and TCS are good (0.49 and 0.23 Å, respectively); visual inspection shows that this zinc atom is biochemically sensible and has a normal binding site. Another zinc atom placed close to the same binding site (from PDB-REDO entry 6a57, ref. ^26^) is marked unreliable based on the local r.m.s.d. value (4.80 Å); the positioning of this zinc ion is most likely incorrect (Fig. 4c).

In the ectonucleotide pyrophosphatase/phosphodiesterase (ENPP) family of proteins a bimetallic zinc site is important for catalysis^27,28^. A structural alignment of the catalytic domain of PDB-REDO models of ENPP1-7 (Fig. 4d) shows that the zinc atoms and residues that coordinate them occupy highly similar positions in all family members. The AlphaFold predictions of the same proteins (AF-P22413, AF-Q13822, AF-O14638, AF-Q9Y6X5, AF-Q9UJA9, AF-Q6UWR7, AF-Q6UWV6 for ENPP1-7, respectively) show more divergence, especially histidine R5 (Fig. 4d). AlphaFill picks up the similarity between the AlphaFold and the PDB-REDO models and transplants both zinc ions into the protein models of ENPPs (Fig. 4d). Histidine R5 having different rotamers in the AlphaFold predictions, which based on the experimental structures should be a single rotamer, suggests that the bimetallic zinc site in the AlphaFill model(s) could benefit from additional refinement.

### Kinases and ATP

**Fig. 5:**
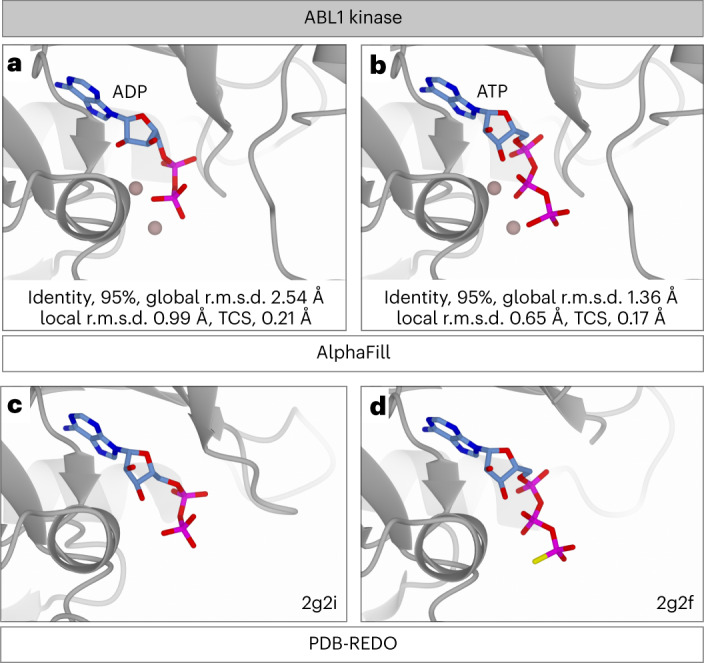
AlphaFill helps to understand the activation state of the Abl kinase AlphaFold model. **a**, AlphaFill model of the ABL1 kinase with ADP and magnesium ions shown. The state of the kinase is not known a priori. **b**, AlphaFill model of the ABL1 kinase with ATP (mapped from AGS) bound. **c**, ADP binding site of the human ABL1 kinase in PDB-REDO entry 2g2i (ref. ^30^), which represents an active kinase state. **d**, ABL1 kinase bound with AGS in PDB-REDO entry 2g2f (ref. ^30^), which represents an ‘intermediate’ kinase state. The kinase is presented as gray ribbon diagram for all panels, ligands are in blue cylinders colored by atom type for noncarbon atoms, and magnesium ions are shown as blush pink spheres.

Kinases are known to have multiple states between the active conformation that offers an environment conducive to the phosphotransfer reaction, and the inactive state that does not fulfill the chemical constraints required for catalytic activity^29^. So far, AlphaFold provides only one conformation per protein. The state to which the AlphaFold models corresponds, is not known a priori. AlphaFill, however, transfers both ADP and ATP (or their analogs) to the AlphaFold model, provided that related experimental structures are available in the PDB-REDO databank, regardless of the functional state of the kinase as characterized by the conformation of specific residues. For the human tyrosine-protein kinase ABL1 (AF-P00519) the AlphaFill model shows an ADP molecule and an ATP molecule (Fig. 5a,b) allowing different hypotheses for the functional state of this model. The global r.m.s.d. for the ADP source is 2.54 and for ATP 1.36 Å, while the local r.m.s.d. for ADP is 0.99 Å and for ATP 0.65 Å. This suggests that the structure is more representative of the ATP-bound state. The AlphaFill entry page informs the user that the ATP molecule was inherited from the ‘B’ chain of the experimental structure 2g2f with bound AGS (ATP-γ-S) (Fig. 5d), an ATP analog that promotes an ‘intermediate’ state in ABL1 (ref. ^30^). Likewise, the ADP has been transplanted from PDB entry 2g2i (ref. ^30^) (Fig. 5c), which represents an active state. Thus, the AlphaFill interface correctly highlights such differences, and allows a simple lookup of the underlying experimental models as well as associated literature to draw relevant conclusions.

## Discussion

Analyzing the contacts of proteins to cofactors, ligands and ions, helps understand both the function and structural integrity of proteins. They can also be helpful for designing downstream experiments, either computational or in the wet laboratory. So far, the AlphaFold database does not include these compounds, but recognizes this need as for each predicted model links to experimental structures are provided through the PDBe-Knowledge Base^12^. Here, we have presented the AlphaFill algorithm to create a resource that takes this further: we do not limit the ‘transplanting’ to the exact same protein, but we extend it to homologs of this model.

The current AlphaFill databank contains transplants of 2,694 different ligands, out of more than 30,000 in the PDB. These represent the most commonly occurring ligands as well as all the cofactors in CoFactor database, and cover about 95% of the cumulative occurrence of ligands in the PDB. We note, that the AlphaFill software is freely available (under the BSD license), which allows users to ‘submit’ any structural model for evaluation, and also the possibility to consider all >30,000 nonpolymer ligands in the PDB. An API to allow users to upload and ‘fill’ their own models or additional structures in the AlphaFold databank (added after June 2022) will be made available, also providing access to additional nonpolymer compounds from the PDB. We note, that currently AlphaFill does not handle polymer ligands, such as peptides, nucleic acids or sugars. It also does not handle posttranslational modifications and, in particular, glycosylation, which is a complicated matter that requires special attention^31^. Other posttranslational modifications such as phosphorylation, frequently induce conformational changes and are likewise not handled in AlphaFill.

An important decision parameter in the AlphaFill algorithm is the minimum sequence identity threshold to allow transfer of information from an experimental structure to an AlphaFold model. We superpose all experimental structures that showed more than 25% sequence identity with AlphaFold models, which also have an alignment length of at least 85 amino acids. This threshold is close to the minimal sequence identity requirement for structural homology^32^. We note that based on our experience with homology restraints^8^ and homology-based annotation of experimental structures^33^ that a threshold closer to 70% is much more reliable for structural details such as local residue interactions; this threshold was also reflected in the validation analysis we present here (Fig. 1c). To allow users to explore possibilities, we have introduced a selector in the web interface that sets the display to the desired identity level on a per-structure basis.

Validation of AlphaFill models against experimental structures with 100% identity, has shown that the local r.m.s.d. and the TCS are good indicators for the reliability of a transplant. A clear color coding to draw the user’s attention to potentially erroneous transfers, indicating medium- and low-confidence transplants based on statistical distributions of these two criteria is used. We also offer the users to run on-the-fly energy minimization to optimize a particular complex of interest. We envisage that users will inspect choices, make selections and then optimize and download the optimized structures most relevant for their research.

The global r.m.s.d. is not a good indicators of transplant quality, but is useful to get a feeling of the similarity between the donor and acceptor structures: a structure with lower global r.m.s.d. but the same or similar identity, denotes a similar conformation. This is reflected in the kinase examples (Fig. 5). We also note that, for multi-domain proteins, the sequence alignment could span all structural domains, but the relative position of each domain might be different in the experimental structure and the model. In this case, the structural alignment may have inflated global r.m.s.d. values due to different relative domain positions. This was observed in the Zn transfer for zinc-finger protein 91 (Fig. 4c).

The AlphaFill structure models are not meant to be accurate or precise or complete representations of the full repertoire of ligands for a certain protein structure. They are meant as a tool for the nonexpert to help them explore complexes with common ligands. Structural biology or structural bioinformatics experts would find it trivial to select, superpose and ‘transplant’ a functional or structural cofactor or ion and take that information to be validated by molecular dynamics simulations and mutagenesis studies, or use it for discussing the structure of a model in light of new biochemical or biophysical insights.

It is good to keep in mind that the AlphaFill models are not very suitable for precise quantification of interactions between the transferred ligand(s) and the protein (for example, hydrogen bonds, π–π or cation–π interactions, van der Waals interactions, hydrophobic interactions, halogen bonds). Namely, this requires coordinate precision that is not provided by either the AlphaFold or the AlphaFill models (even after optimization). Hence, the models should be interpreted in a qualitative manner. Moreover, in some cases ligand interactions involve parts of the protein that are not modeled with high confidence by AlphaFold; while optimization might improve the local environment, we advise caution.

Besides using several optimized and robust defaults, the AlphaFill software is made to be flexible by design so that the used settings and cutoffs can easily be tailored to any user’s own purposes. Similarly, the list of transferrable compounds can readily be updated based on user requirements; we invite users to provide constructive feedback to allow to further develop these services.

AlphaFill by definition depends on high-quality structure homologs as the first and main criterion for transferring ligands. However, it is well established that certain structural domains can occur outside the context of extensive sequence similarity as it has been shown for example by DALI^34^ and PDBeFold^35^. Thus, AlphaFill could be complemented by structure-based transfer algorithms based on deep learning concepts similar to those used for the AlphaFold structure prediction revolution.

## Methods

### Detailed overview of the procedure

The AlphaFill procedure for filling up missing information to AlphaFold models goes through the following steps.

1. The amino-acid sequence of each AlphaFold model is BLASTed^45^ against the sequence file of the LAHMA webserver^33^, which contains all sequences present in the PDB-REDO databank. The alignments, that is individual high-scoring segment pairs (HSPs) are sorted by *E* value to capture both the sequence similarity and the length of the alignment as they are combined factors in conferring structural homology. A maximum of 250 hits, as is the default for BLAST, is returned.
2. The structure models corresponding to these hits are retrieved from the PDB-REDO databank and checked for compounds of interest for the AlphaFill algorithm (vide infra).
3. The hits with compounds of interest are filtered to ensure that only sufficiently close homologs are used. Currently, we use a sequence identity cutoff of 25% over an aligned HSP of at least 85 residues. For such an alignment length, identities as low as 25% still confer overall structural homology^32^.
4. This selection of hits is structurally aligned^11^ on the Cα-atoms of the residues that match in the BLAST alignment. The r.m.s.d. of this global alignment is stored in the AlphaFill metadata. Note that a single PDB-REDO model chain can have several HSPs. These are aligned individually.
5. Starting from the hit with the smallest BLAST *E* value, each compound of interest in the hit list is scanned for its local surroundings. All backbone atoms within 6 Å are then used for a local structural alignment to the AlphaFold model. The r.m.s.d. of this local alignment is also stored in the AlphaFill metadata.
6. Compounds are then integrated into the AlphaFold model to make its AlphaFill counterpart, unless the same compound has already been placed within 3.5 Å of the centroid of the compound to be fitted (originating from a previously considered homolog) or no protein atoms are present within 4.0 Å from the atoms of the compound to be fitted. If compounds have multiple conformations, all of these are included in the AlphaFill model. Descriptions of covalent bonds or metal binding captured in so-called struct_conn records are also added to the AlphaFill model.
7. For each transplant a TCS is calculated using equation (1) and stored in the metadata. The TCS is the r.m.s. van der Waals overlap over all atomic distances between the transplant atoms and the protein that are shorter than 4 Å.
  1 $${\mathrm{TCS}} = \sqrt {\frac{{{\mathrm{vd}}\ {\mathrm{Waals}}\ {\mathrm{overlap}}_i^2 + {\mathrm{vd}}\ {\mathrm{Waals}}\ {\mathrm{overlap}}_j^2 + {\mathrm{vd}}\ {\mathrm{Waals}}\ {\mathrm{overlap}}_k^2 + \ldots }}{{{\mathrm{Number}}\,{\mathrm{of}}\,{\mathrm{distances}}\,{\mathrm{considered}}}}}$$
8. The AlphaFill model with all transplanted compounds is finally stored as mmCIF coordinate file together with a JSON-formatted metadata file describing the provenance of each transplanted compound.

The running time per model depends strongly on the number of BLAST hits and compounds to be transferred. The mean running time is 2 minutes per model on a single CPU thread.

### Input data: protein structure models

All AlphaFold models^1^ (available 1 February 2021) were downloaded from the AlphaFold Protein Structure Database’s FTP archive. A local copy of the PDB-REDO databank^8^ was used to provide ligands for transfer.

To find all relevant PDB-REDO entries for a specific AlphaFold model through sequence-based retrieval with BLAST, a PDB-REDO-specific sequence database (as of 1 February 2021) was used. This database is created automatically as part of the weekly LAHMA and PDB-REDO databank updates.

### Input data: selection of chemical compounds

We decided to only consider compounds that likely represent common biological states and are likely suited for further study. Thus, a collection of common biologically relevant cofactors, ligands and metal ions was created.

The selection of biological relevant ligands to be added to the AlphaFold models was performed based on the number of their occurrences in the PDB. All ligands covering about 95% of the cumulative occurrence of all ligands in the PDB were in the initial AlphaFill compound list that was complemented with all cofactors and their analogs present in the organic CoFactor database^9^ that were not within the 95% cumulative occurrence. To map cofactor analogs and adducts to their canonical cofactors where possible, analogs were mapped to their representative cofactor by atom renaming (and atom deletion); for example, adenosine-5′-(beta,gamma-methylene)triphosphate (methylene substituted ATP) is translated to ATP, as ATP is the compound involved in biological processes. Cofactor adducts such as CNC (vitamin B12 in complex with cyanide) are trimmed down to their parent (for example, vitamin B12 in the CNC case) by atom deletion. Cofactor analogs that have atoms missing with respect to their parent are kept as is. The required changes were found by visual inspection of the compounds via the Ligand-Expo website^46^ and the PDB web sites. Common crystallization agents (for example, poly-ethyleneglycol and chloride), some metals with unclear physiological importance (for example, cadmium ions), posttranslational modifications (modified amino acids) and other polymers (peptides, nucleic acids and carbohydrates) were purposely excluded. All information was stored in a CIF-formatted data file that can easily be extended.

The current collection of compounds to be transplanted consists of 2,694 entries. It is stored separate from the AlphaFill program to allow easy extension in future incarnations of the AlphaFill databank and is freely available.

### The AlphaFill software

A new program, AlphaFill, was created for the purpose of this study. AlphaFill reads an AlphaFold model together with the compound list and the PDB-REDO-specific sequence database and structures, and returns a structure model consisting of the coordinates of the AlphaFold model plus all transferred compounds. See above for the compound transfer procedure. The AlphaFill program is based on the libzeep^47,48^, libcif++ (ref. ^49^) (a general purpose C++ library for dealing with mmCIF data structures), libpdb-redo (a core library for PDB-REDO software) and clipper^50^ libraries, and contains its own BLAST implementation. The source codes of AlphaFill, libcif++ and libpdb-redo are available from https://github.com/PDB-REDO.

### Creation of the AlphaFill databank

The AlphaFill databank was created by running AlphaFill over all AlphaFold models. The computational workload is parallel that allows orchestration of the calculations by using the software make^51^, as we have done previously^52^, with the AlphaFold coordinate files as sources and the AlphaFill coordinate files as targets. The calculation took 15 days on a server with a total of 90 CPU threads.

### The AlphaFill web interface

The web site was created as a web application using the libzeep library that offers an HTTP server, HTML templating and many other components for web server construction in C++. Handling of mmCIF files is done using libcif++. The data for the Models, Structures and Compounds pages are stored in a PostgreSQL^53^ database. The model is presented on the page using Mol*^16^ as an interactive web component.

### Validation of the AlphaFill algorithm

To validate the AlphaFill algorithm, all transplanted compounds that were obtained from a donor PDB-REDO model with 100% sequence identity were selected as validation set (28,619 transplants). For each compound in this set, we calculated the all-atom r.m.s.d. with respect to the donor model for the transplant binding site that we called the LEV score. The transplant binding site consists of all nonhydrogen atoms of the transplant and all nonhydrogen protein atoms within 6.0 Å of the transplant atoms.

The LEV score was correlated to the local r.m.s.d. and to the TCS, which are both calculated in the AlphaFill algorithm for each transplant. The Pearson correlation coefficient was calculated using DataFrame.corr() in pandas v.1.2.4.

### Model refinement

The AlphaFill web interface allows the refinement of individual transplants in the context of the protein. When a single transplant is selected, a user can activate its refinement. A new structure file containing only the protein and the selected transplant is created and passed to the refinement engine that runs on the server backend. The refinement procedure is based on the ‘Energy minimization’ experiment in YASARA^13^ that consists of a steepest descent minimization followed by a short simulated annealing in the updated YASARA NOVA^54^ force field. All default settings are used and forcefield parameters for the transplant are generated on-the-fly by YASARA. After the energy minimization, the TCS of the transplant is recalculated. The original and new TCS values are displayed together with a Mol* viewer of the refined model. The refined model can also be downloaded.

### Validation of the refinement procedure

The refinement engine provides the option to energy minimize a specific transplant in complex with the protein on demand. To validate the refinement results, the TCS and LEV score before and after refinement were obtained and analyzed for four subsets of compounds in the validation set: (1) the 50 lowest TCS, (2) the 50 transplants with TCS closest to 0.25 Å, (3) the 50 transplants with TCS closest to 0.50 Å and (4) the 50 transplants with the highest TCS.

### Model and data analysis

The AlphaFill models were analyzed visually using Coot^55^, the AlphaFill website and CCP4mg (ref. ^56^). Plots were made using Seaborn^57^, molecular graphics figures were made with CCP4mg. Data analyses for validation were performed using Python v.3.7.9 with the numpy v.1.20.3 and pandas v.1.2.4 packages.

### Reporting summary

Further information on research design is available in the Nature Portfolio Reporting Summary linked to this article.

## Online content

Any methods, additional references, Nature Portfolio reporting summaries, source data, extended data, supplementary information, acknowledgements, peer review information; details of author contributions and competing interests; and statements of data and code availability are available at 10.1038/s41592-022-01685-y.

## Supplementary information

Supplementary Information Supplementary Figs. 1 and 2.

Reporting Summary

## Data Availability

All input data used in this study are freely available from PDB-REDO (https://pdb-redo.eu), AlphaFold (https://alphafold.ebi.ac.uk/) and CoFactor (http://www.ebi.ac.uk/thornton-srv/databases/CoFactor/). All data discussed in this paper are publicly available from https://alphafill.eu. An individual AlphaFill entry (entryid) can be downloaded via the graphical user interface. In addition, structure files in mmCIF format are available for every entry at: https://alphafill.eu/v1/aff/${entryid}. JSON files with the metadata for the transplants are available at: https://alphafill.eu/v1/aff/${entryid}/json. The JSON schema providing details on the metadata is at https://alphafill.eu/alphafill.json.schema. The complete AlphaFill databank can be freely downloaded by the command: rsync -av rsync://rsync.alphafill.eu/alphafill {destination folder}/.

## Code availability

The AlphaFill code used for this study is available through Zenodo at https://zenodo.org/record/6706668#.Y2EXV3bP2Uk. Current and future versions are open source with a BSD-2-clause license and available from https://github.com/PDB-REDO/alphafill.
