# Supplementary material for: AlphaFill: enriching AlphaFold models with ligands and cofactors
Source: Nat Methods. 2022 Nov 24;20(2):205–13. doi: 10.1038/s41592-022-01685-y (PMC9911346; doi:10.1038/s41592-022-01685-y)
Supplement: Supplementary file 1 — Supplementary Figs. 1 and 2. [file 41592_2022_1685_MOESM1_ESM.pdf]

nature methods

Resource https://doi.org/10.1038/s41592-022-01685-y

AlphaFill: enriching AlphaFold models with ligands and cofactors

In the format provided by the authors and unedited

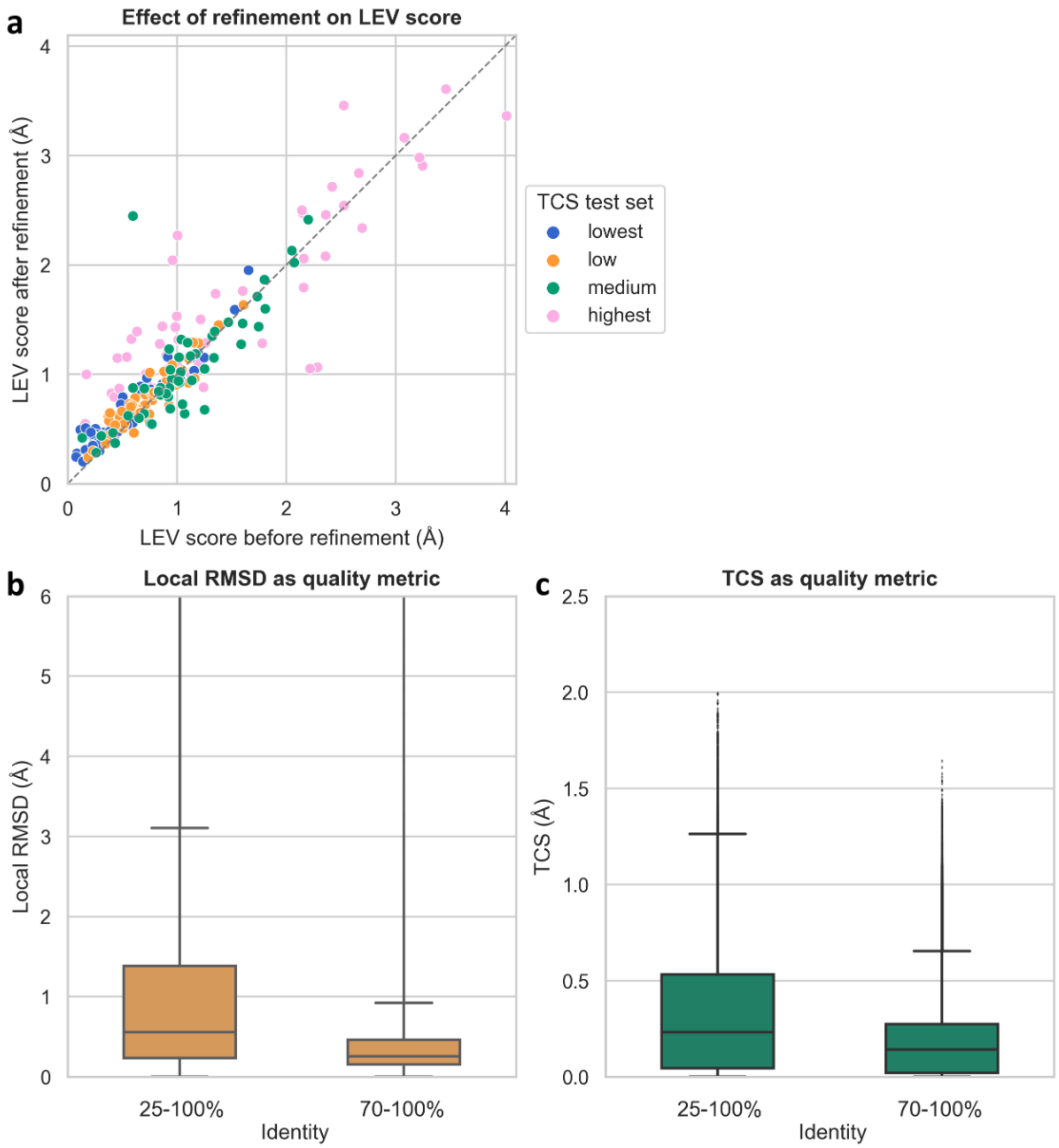

Supplemental Figure S1: **a** Comparison of the LEV score before and after energy minimisation for four subsets of the validation set (n = 50 for each subset), illustrating that LEV score is not strongly affected for low(est) TCS, whereas high(er) initial TCS shows some changes upon refinement. **b** Local RMSD boxplots that were used to determine the cut-offs to annotate high, medium and low-confidence transplants. Boxes are based on 12,029,789 and 933,117 transplants and extend from first to third quartile with the median as the middle line. Whiskers extend to 1.5 times the inter-quartile range. Outliers are shown as individual points. 466,867 and 3,373 outliers not shown for clarity, maximum values are

107.4 Å and 44.4 Å. **c** TCS boxplots that were used to determine the cut-offs to annotate high, medium and low-confidence transplants. Boxes are based on 12,029,789 and 933,117 transplants and extend from first to third quartile with the median as the middle line. Whiskers extend to 1.5 times the inter-quartile range. Outliers are shown as individual points.

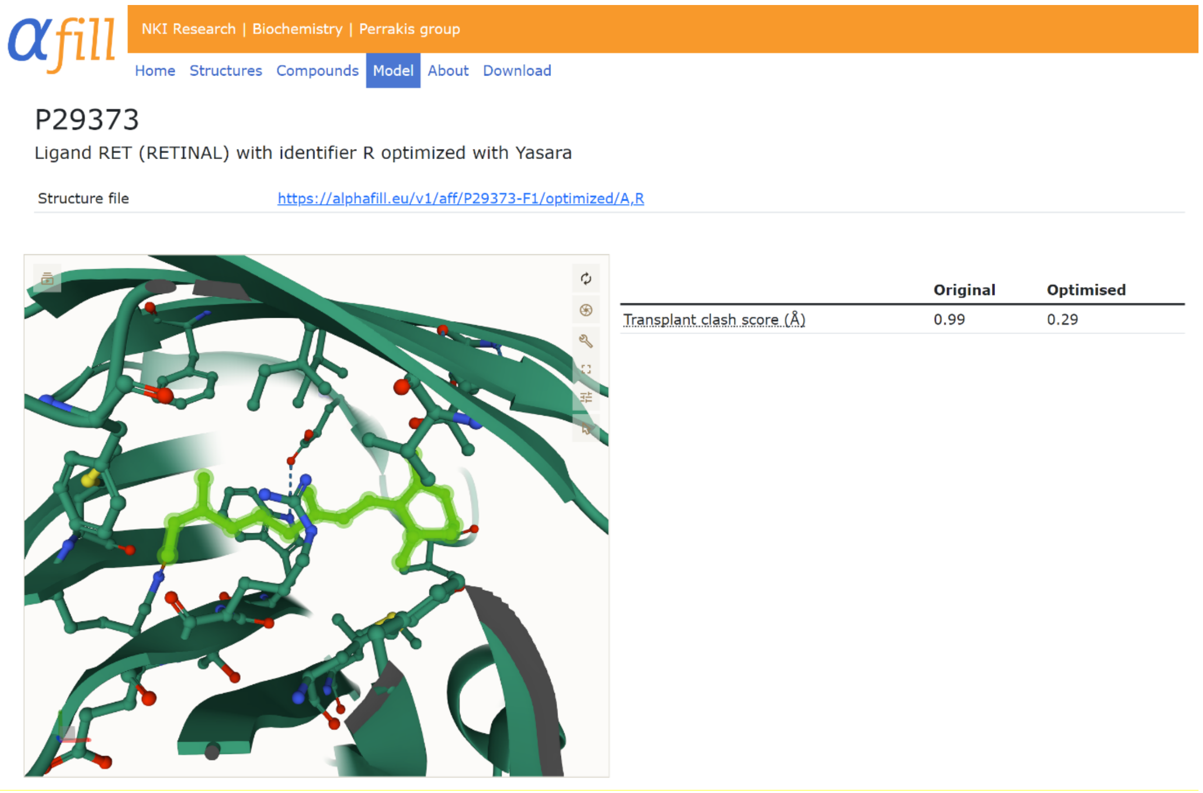

Supplemental Figure S2: Screenshot after using the “optimise” function for retinal (RET) in cellular retinoic acid-binding protein 2 (AF-P29373). The TCS score before and after refinement is shown, along with a ligand-focused view in Mol*. The optimised complex can be downloaded.
