# Supplementary material for: AlphaFill: enriching AlphaFold models with ligands and cofactors
Source: Nat Methods. 2022 Nov 24;20(2):205–13. doi: 10.1038/s41592-022-01685-y (PMC9911346; doi:10.1038/s41592-022-01685-y)
Supplement: Supplementary file 2 — Reporting Summary [file 41592_2022_1685_MOESM2_ESM.pdf]

nature research

Corresponding author(s): Anastassis Perrakis (a.perrakis@nki.nl), Robbie P. Joosten (r.joosten@nki.nl)

Last updated by author(s): Sep 5, 2022

Reporting Summary

Nature Research wishes to improve the reproducibility of the work that we publish. This form provides structure for consistency and transparency in reporting. For further information on Nature Research policies, see our Editorial Policies and the Editorial Policy Checklist.

Statistics

For all statistical analyses, confirm that the following items are present in the figure legend, table legend, main text, or Methods section.

n/a	Confirmed	
☐	☒	The exact sample size (*n*) for each experimental group/condition, given as a discrete number and unit of measurement
☒	☐	A statement on whether measurements were taken from distinct samples or whether the same sample was measured repeatedly
☒	☐	The statistical test(s) used AND whether they are one- or two-sided<br>*Only common tests should be described solely by name; describe more complex techniques in the Methods section.*
☒	☐	A description of all covariates tested
☒	☐	A description of any assumptions or corrections, such as tests of normality and adjustment for multiple comparisons
☐	☒	A full description of the statistical parameters including central tendency (e.g. means) or other basic estimates (e.g. regression coefficient) AND variation (e.g. standard deviation) or associated estimates of uncertainty (e.g. confidence intervals)
☒	☐	For null hypothesis testing, the test statistic (e.g. *F*, *t*, *r*) with confidence intervals, effect sizes, degrees of freedom and *P* value noted<br>*Give P values as exact values whenever suitable.*
☒	☐	For Bayesian analysis, information on the choice of priors and Markov chain Monte Carlo settings
☒	☐	For hierarchical and complex designs, identification of the appropriate level for tests and full reporting of outcomes
☐	☒	Estimates of effect sizes (e.g. Cohen's *d*, Pearson's *r*), indicating how they were calculated

Our web collection on statistics for biologists contains articles on many of the points above.

Software and code

Policy information about availability of computer code

Data collection	No specific data collection software was used. The AlphaFill code used for this study is available through Zenodo with DOI: 10.5281/zenodo.6706668. Current and future versions are open source with a BSD-2-clause license and available from https://github.com/PDB-REDO/alphafill.
Data analysis	Data analyses for validation were performed using Python3.7.9 with the numpy version 1.20.3 and pandas version 1.2.4 packages. Visual examination of models was performed with Coot versions 0.8.9.2 to 0.9.8.3.

For manuscripts utilizing custom algorithms or software that are central to the research but not yet described in published literature, software must be made available to editors and reviewers. We strongly encourage code deposition in a community repository (e.g. GitHub). See the Nature Research guidelines for submitting code & software for further information.

Data

Policy information about availability of data

All manuscripts must include a data availability statement. This statement should provide the following information, where applicable:

- Accession codes, unique identifiers, or web links for publicly available datasets
- A list of figures that have associated raw data
- A description of any restrictions on data availability

All input data used in this study are freely available from PDB-REDO (https://pdb-redo.eu), AlphaFold (https://alphafold.ebi.ac.uk/), and CoFactor (http://www.ebi.ac.uk/thornton-srv/databases/CoFactor/).
All data created and discussed in this paper are publicly available from https://alphafill.eu. An individual AlphaFill entry (entryid) can be downloaded via the graphical user interface. In addition, structure files in mmCIF format are available for every entry at: “https://alphafill.eu/v1/aff/${entryid}”. JSON files with the meta-data for the transplants are available at: “https://alphafill.eu/v1/aff/${entryid}/json”. The JSON-schema providing details on the metadata is at “https://

alphafill.eu/alphafill.json.schema". The complete AlphaFill databank can be freely downloaded by the command: "rsync -av rsync://rsync.alphafill.eu/alphafill {destination folder}/".
The following license applies:
"Data files contained in the AlphaFill databank (rsync://rsync.alphafill.eu; https://alphafill.eu) are fully and freely available for both non-commercial and commercial use. Users of the data should attribute both AlphaFill and AlphaFold. By using the materials available in the AlphaFill, the user agrees to abide by the conditions described below:

* The archival data files in the AlphaFill archive are made freely available to all users. Data files within the archive may be redistributed in original form without restriction. Redistribution of modified data files is allowed only if the parent data file is attributed.

* Where applicable, the usage policy of the parent AlphaFold archive entries applies.

* The data in the AlphaFill databank are provided on an "as is" basis. Neither AlphaFill nor its parent or comprising institutions can be held liable to any party for direct, indirect, special, incidental, or consequential damages, including lost profits, arising from the use of AlphaFill materials.

* Resources on alphafill.eu are provided without warranty of any kind, either expressed or implied. This includes but is not limited to merchantability or fitness for a particular purpose. The institutions managing this site make no representation that these resources will not infringe any patent or other proprietary right.

Field-specific reporting

Please select the one below that is the best fit for your research. If you are not sure, read the appropriate sections before making your selection.

☒ Life sciences ☐ Behavioural & social sciences ☐ Ecological, evolutionary & environmental sciences

For a reference copy of the document with all sections, see nature.com/documents/nr-reporting-summary-flat.pdf

Life sciences study design

All studies must disclose on these points even when the disclosure is negative.

Sample size	The sample consisted of all AlphaFold entries (February 2022), n = 995411
Data exclusions	No data were excluded
Replication	The study was computational and non-stochastic which made replication unnecessary. That is, any repeat calculation with the same input will return exactly the same results.
Randomization	Rather than performing calculations on random subsets of the AlphaFold Databank, the entire data set was considered. Therefore randomisation was not applicable as there is no sampling.
Blinding	The study was purely computational and contained no aspects where the use of blinding has any effect on the outcome of the performed calculations.

Reporting for specific materials, systems and methods

We require information from authors about some types of materials, experimental systems and methods used in many studies. Here, indicate whether each material, system or method listed is relevant to your study. If you are not sure if a list item applies to your research, read the appropriate section before selecting a response.

Materials & experimental systems

n/a	Involved in the study	
☒	☐	Antibodies
☒	☐	Eukaryotic cell lines
☒	☐	Palaeontology and archaeology
☒	☐	Animals and other organisms
☒	☐	Human research participants
☒	☐	Clinical data
☒	☐	Dual use research of concern

Methods

n/a	Involved in the study	
☒	☐	ChIP-seq
☒	☐	Flow cytometry
☒	☐	MRI-based neuroimaging
